# The complete mitochondrial genome of the *Hieroglyphus tonkinensis* (Orthoptera: Acrididae)

**DOI:** 10.1080/23802359.2016.1197067

**Published:** 2016-07-23

**Authors:** Huihui Chang, Yuan Huang

**Affiliations:** College of Life Sciences, Shaanxi Normal University, Xi’an, China

**Keywords:** Acrididae, Hieroglyphus tonkinensis, Mitogenome, phylogeny

## Abstract

*Hieroglyphus tonkinensis* (Orthoptera: Caelifera: Acrididae) is an important agricultural pest to bamboo, rice and other gramineous crops. The complete mitochondrial genome of *H. tonkinensis* is 15,625 bp in length and consists of 13 protein-coding genes, 22 tRNA genes, 2 rRNA genes and 1 A + T-rich region. The gene order of the mitogenome is identical with most orthopteran insects. Most protein-coding genes start with typical ATN codon except for *COX1*, which initiates with ACC codon instead. While all PCGs use complete stop codons (TAA and TAG). In addition, 13 related species and 2 outgroup taxa were used to construct the phylogenetic tree to further validate the mitogenome of *H. tonkinensis*. The result showed that *H. tonkinensis* is sister group to a clade of *Oxya* and *Pseudoxya*.

*Hieroglyphus tonkinensis* (Orthoptera: Caelifera: Acrididae) with gregariousness, flying force, move quickly, jumping strong, omnivorous and other characteristics, is a pest of bamboo, cane, rice, wheat, corn and other cereal crops. The insect is mainly distributed in Taiwan, Guangdong, Guizhou, Fujian, Hainan provinces and Guangxiautonomous regions in China (Lu et al. 2011). According to the Orthoptera Species File Online, Acrididae, as a subfamily, is included in the Acridoidea (Eades et al. 2015).

Mitochondrial DNA, as an effective molecular marker, is commonly used to investigate the population structure, phylogeography and phylogenetic analyses of insects (Ma et al. 2012; Zhang et al. 2013). However, the mitochondrial genome of *H. tonkinensis* is not available at present. Specimens of H. tonkinensis were collected from LiuwanDashan in Yulin City (Guangxi, China; N22°33′N, 109°46′E). the specimens were preserved in 100% absolute ethanol and stored in −20 °C freezer in Institute of Zoology of Shaanxi Normal University (accession No. M0683). Total genomic DNA was extracted from the muscle of one of the specimen’s femurs by the standard proteinase K and phenol/chloroform extraction method, then stored at −20 °C. Here, we report a complete mitochondrial genome of *H. tonkinensis*, which is 15 625 bp in length and has been deposited in GenBank (accession No. KX170936). It contains 13 typical protein-coding genes, 22 tRNA genes, 2 rRNA genes and 1 A + T-rich region, which is like the other orthopteran insects. The gene arrangement was identical with *Calliptamus italicus* (GenBank accession No.: NC_011305; Fenn et al. 2008). The overall base composition of the whole mitochondrial genome was A (42.9%), T (31.3%), C (15.1%) and G (10.8%), with an AT bias of 74.2%, as generally reported in other orthopteran mitogenomes. The 22 tRNA genes which were predicted by online software tRNAScan-SE (Lowe & Eddy 1997), rank from 62 bp (*tRNA^Cys^*) to 72 bp (*tRNA^Val^*) in length. Gene overlaps in the mitogenome of *H. tonkinensis* in a total of 41 bp in six locations with length in 1 ∼ 8 bp. The longest overlaps occur between *trnW*/*trnC* and *trnY*/*COX1*. The mitogenome has 21 intergenic spacers in a total of 146 bp with length varying from 1 to 35 bp.

All tRNAs except *tRNA^Ser(AGN)^* could be folded into typical cloverleaf secondary structures. Two rRNA genes were 1322 bp (*lrRNA*) and 795 bp (*srRNA*) long, which are located between *tRNA^Leu(CUN)^* and A + T-rich region, separated by *tRNA^Val^.* Only *COX1* uses ACC as a start codon, but the other protein-coding genes begin with the canonical ATN codon. While all the 13 PCGs use complete stop codons (TAG and TAA). The A + T-rich region, which is located between *srRNA* and *tRNA^Ile^*, is 774 bp in length and has 85.3% AT content.

To further validate the mitogenome of *H. tonkinensis*, the phylogenetic analyses were performed using MrBayes 3.1.2 (Ronquist & Huelsenbeck 2003) based on the concatenated dataset (PCGs) of mitogenomes of *H. tonkinensis* and the other 15 taxa that were retrieved from GenBank, including 13 Acrididae ingroup and two outgroup taxa (Figure 1).The result showed that *H. tonkinensis* is sister group to a clade of *Oxya* and *Pseudoxya*.

**Figure 1.**
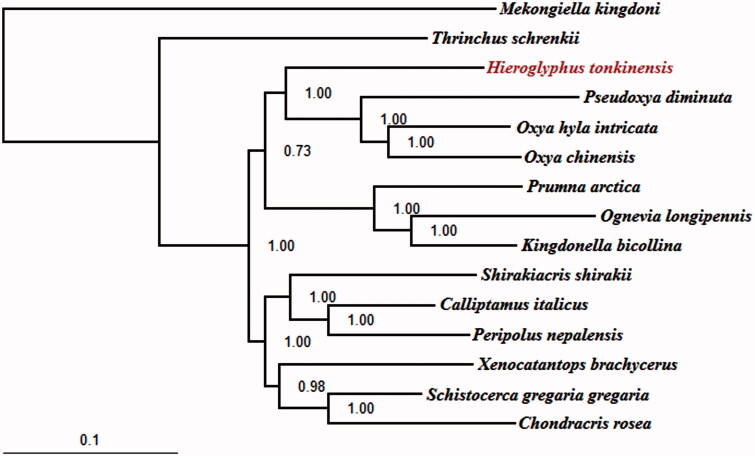
The BI phylogenetic tree of the Hieroglyphus tonkinensis in this study and other 13 related species and 2 outgroup taxa based on mitochondrial PCGs concatenated dataset. Note: Mekongiella kingdoni: NC_023921; Thrinchus schrenkii: NC_014610. The 2 outgroup taxa belong to Acridoidea. Hieroglyphus tonkinensis: KX170936; Pseudoxya diminuta: NC_025765; Oxya hyla intricata: KP313875; Oxya chinensis: NC_010219; Prumna arctica: NC_013835; Ognevia longipennis: NC_013701; Kingdonella bicollina: NC_023920; Shirakiacris shirakii: NC_021610; Calliptamus italicus: NC_011305; Peripolus nepalensis: NC_029135; Xenocatantops brachycerus: NC_021609; Schistocerca gregaria gregaria: NC_013240; Chondracris rosea: NC_019993. The 13 related species all belong to Acrididae.
